# The association between HER2-low status and survival in patients with metastatic breast cancer treated with Cyclin-dependent kinases 4 and 6 inhibitors: a systematic review and meta-analysis

**DOI:** 10.1007/s10549-023-07226-1

**Published:** 2024-01-19

**Authors:** Deniz Can Guven, Taha Koray Sahin

**Affiliations:** 1 Hacettepe University Cancer Institute, Ankara, Turkey; 2 Health Sciences University, Elazig City Hospital, Elazig, Turkey; 3 Hacettepe University Internal Medicine, Ankara, Turkey

**Keywords:** HER2-low, HER2-zero, Breast cancer, CDK4/6 inhibitors, Prognosis

## Abstract

**Purpose:**

The cyclin-dependent kinase (CDK) 4/6 inhibitors significantly altered the treatment landscape of hormone-positive (HR+), HER2- metastatic breast cancer (MBC). However, biomarkers predicting long-term benefit and early progression are yet to be defined. Several studies suggested the possibility of diminished efficacy in patients with HER2-low disease. Therefore, we conducted a systematic review and meta-analysis to evaluate the association between low-level HER2 expression and efficacy outcomes (PFS, OS, ORR) with CDK 4/6 inhibitors.

**Methods:**

The Pubmed, Web of Science, and Scopus databases were used to systematically filter the published studies from inception to 08 August 2023 for this systemic review. Studies including MBC patients treated with CDK 4/6 inhibitors and reported survival outcomes according to HER2 expression were included. We performed the meta-analyses with the generic inverse-variance method with a fixed-effects model and used HRs with 95% two-sided CIs as the principal summary measure.

**Results:**

Nine studies encompassing 2705 patients were included in the analyses. In the pooled analysis of nine studies, the risk of progression and/or death was higher in patients with HER2-low tumors compared to HER2-zero (HR: 1.22, 95% CI 1.10–1.35, *p* < 0.001). In the pooled analysis of five studies, although the median follow-up was short, the risk of death was higher in the HER2-low group compared to the HER2-zero group (HR: 1.22, 95% CI 1.04–1.44, *p* = 0.010).

**Conclusion:**

The available evidence demonstrates a significantly higher risk of progression or death with CDK 4/6 inhibitors in HER2-low tumors. Further research is needed to improve outcomes in patients with HR+-HER2-low tumors.

## Introduction

The cyclin-dependent kinase (CDK) 4/6 inhibitors significantly altered the treatment landscape of hormone-positive (HR+), HER2- metastatic breast cancer (MBC) [1–3]. The combination of CDK 4/6 inhibitor plus endocrine therapy became the standard of care option in the first- and second-line settings with improved progression-free (PFS) and overall survival (OS) data [4, 5]. Currently, these agents are being used independent of a biomarker status in clinical scenarios other than visceral crisis, in parallel with pivotal phase III trials [6–8]. However, not all patients uniformly benefit from these treatments, and around 15% of the patients progressed even with first-line use [6, 9]. Therefore, biomarkers predicting long-term benefits and early progression are needed.

The ErbB2 receptor family plays a pivotal role in endocrine treatment resistance, and targeted therapies to this pathway have been used over two decades in HER2+breast cancer [10]. The HER2+tumors are classified as tumors with a 3+IHC or 2+IHC and ISH positivity. Considering the lower levels of HER2 expression in HER2 1 + or HER2 2 + and ISH-negative tumors and the possibility of targeting these tumors with novel anti-HER2 drug antibody conjugates [11, 12], we witnessed the emergence of a new subgroup of breast tumors called “HER2-low breast cancer” [13, 14]. However, the effects of low-level HER2 expression on the survival are yet to be defined [15, 16]. While some studies reported inferior survival in patients with HR+HER2-low tumors, several studies stated similar survival in HER2-low and HER2-zero tumors [17–19]. In addition to the prognosis, the low levels of HER2 expression could affect the efficacy of anti-endocrine agents, including the CDK 4/6 inhibitors, due to the pivotal role of the ErbB2 receptor on endocrine resistance [20]. However, the available studies differed in study designs, patient populations, sample sizes, as well as outcomes. Therefore, we conducted a systematic review and meta-analysis to evaluate the prognostic role of low-level HER2 expression on the outcomes of MBC patients treated with CDK 4/6 inhibitors.

## Material and methods

### Literature search

We conducted a systematic review following the Preferred Reporting Items for Systematic Reviews and Meta-analysis guidance (PRISMA) [21]. The study protocol was registered with the PROSPERO (CRD42023453557). The Pubmed, Web of Science, and Scopus databases were used to systematically filter the published studies from inception to August 08, 2023, for this systemic review. The selected MeSH search terms were “HER2 low” OR “low HER2” OR “ERBB2 low” OR “low ERBB2” AND “CDK” OR “cyclin-dependent kinase” OR “CDK 4/6” OR “CDK4/6” OR “CDK 4/6 inhibitor.”

### Inclusion and exclusion criteria

We included studies that met the following inclusion criteria: (1) prospective or retrospective study to evaluate the potential association of low-level HER2 expression on either progression-free survival (PFS) or overall survival (OS) with CDK 4/6 inhibitors; (2) available hazard ratio and 95% confidence interval for the comparison of HER2-low and HER2-zero groups; and (3) peer-reviewed full-text article or abstract available in English. Exclusion criteria of studies were: (1) duplicated articles; (2) review articles, case reports, case series, editorials, guidelines, dissertations, and opinion papers; (3) animal and cell-line studies; (4) studies including pediatric patients; (5) studies comparing HER2-positive and HER2-negative patients; (6) studies reporting on outcomes other than PFS or OS, and (7) trial protocols.

### Study selection and data extraction

**Fig. 1:**
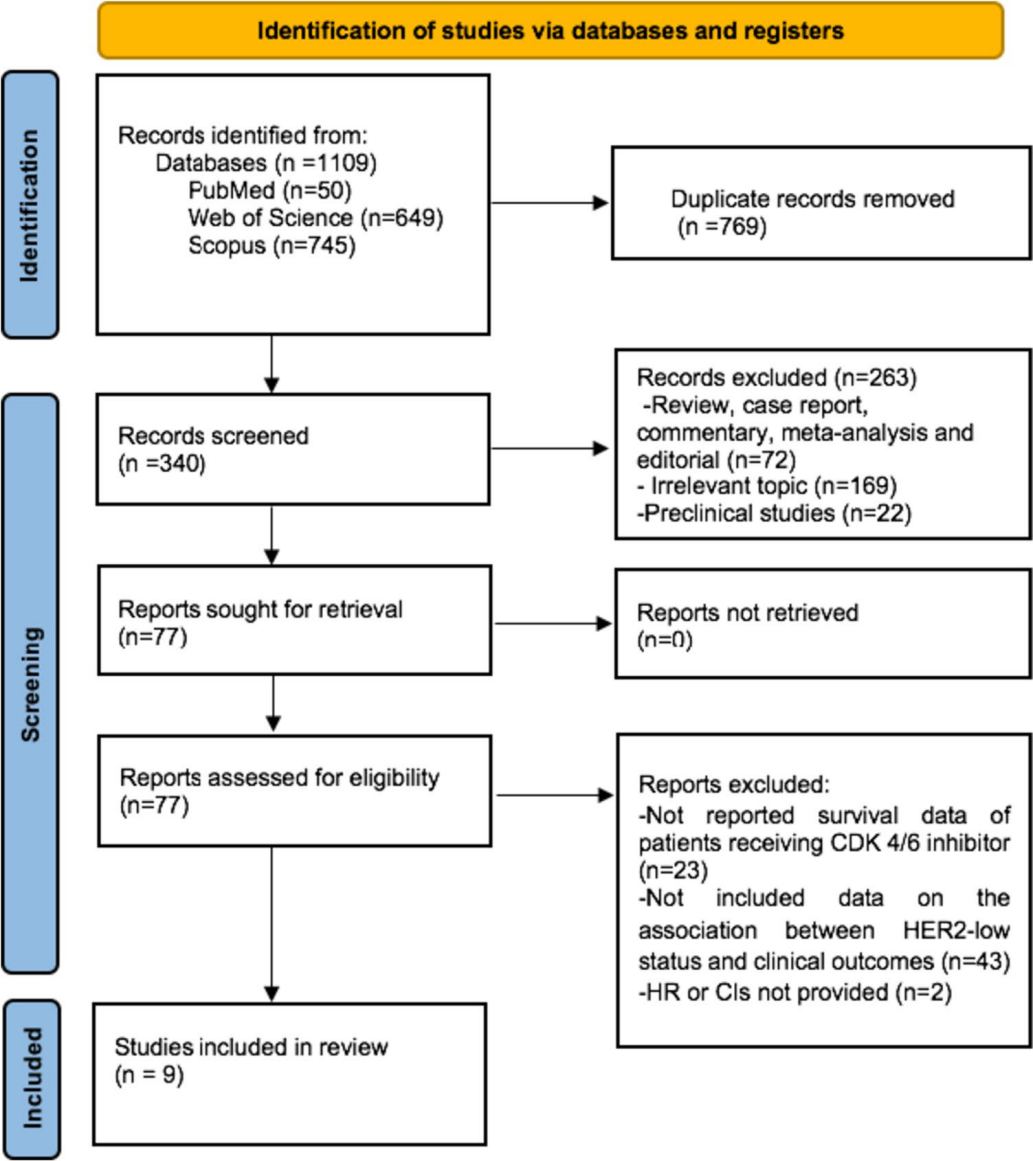
PRISMA flow diagram

Our systematic search retrieved 1109 records. After removing duplicates (*n* = 769), we screened the remaining 340 records for inclusion. A total of 263 records were excluded after the screening of titles and abstracts. After evaluation of the full texts of the remaining 77 records, we excluded 67 more records due to no survival data (*n* = 23), no data on the association between low HER2 status and survival outcomes (*n* = 43), and no available HR or CI (*n* = 2); and included nine studies from the systematic search in meta-analyses. The flowchart for article selection is shown in Fig. 1.

Two authors (DCG, TKS) extracted the data following the Meta-analysis of Observational Studies in Epidemiology (MOOSE) guidelines and any discrepancy was resolved by the senior author [22]. The following data were extracted from the available studies: lead author names, year of publication, total number of patients, hazard ratios (HR) with 95% CIs for OS or PFS, and overall response rate (ORR). The individual study qualities and risk of bias were evaluated independently by two authors (DCG and TKS) using the Newcastle–Ottawa Scale.

### Meta-analysis

The primary objective of this study was to evaluate the association between PFS and low levels of HER2 expression in patients with HR+breast cancer treated with CDK 4/6 inhibitors. The secondary objective was to evaluate the association between the OS and ORR according to HER2 expression (HER2 low vs. HER2 zero. We conducted further subgroup analyses for PFS according to the treatment line.

We performed the meta-analyses with the generic inverse-variance method with a fixed-effects model, considering the low degree of heterogeneity in the analyses. We used HRs with 95% two-sided CIs as the principal summary measure and reported the heterogeneity within each subgroup with I-square statistics. We conducted the meta-analyses using the Review Manager software, version 5.4 (The Nordic Cochrane Center, The Cochrane Collaboration, Copenhagen, Denmark) and considered p values below 0.05 statistically significant.

## Results

### Study characteristics

Nine studies encompassing a total of 2705 patients were included in the analyses. The four studies were conducted in the first line [18, 23–25], while mixed cohorts were present in five studies [17, 19, 26–28]. Five studies were multicenter, and single-center data were reported in four studies. Eight studies were retrospective, while only one included a cohort with prospectively recorded data. All studies included both patients treated with aromatase inhibitors or fulvestrant in combination with CDK 4/6 inhibitors. Sample sizes varied between 84 and 1084, and five of nine studies had sample sizes of less than 200 patients. Four of the studies were from Europe. The PFS and OS were available in five studies, while four studies reported only PFS. The median follow-up time varied between 15 and 36 months across studies (Table 1). Most studies had a low risk of bias, according to the NOS (Table 2).

**Table 1:** Characteristics of included studies

Author, year	Country	Type of Study	Total number of patients	Number of Patients (HER2-Low/Zero)	Median age, year	Line of therapy	Treatment	Median OS (HER2-Low vs HER2-Zero)	Median PFS (HER2-Low vs HER2-Zero)	ORR (HER2-Low vs HER2-Zero)	Median follow-up, mo
Bao, 2021 [26]	Hong Kong	Single-center Retrospective	106	82/24	58	Mixed	CDK4/6 inhibitor (Palbociclib/ribociclib)+AI or Fulvestrant	N/A	8.9 vs. 18.8 mo	N/A	N/A
Bortot, 2021 [23]	Italy	Multicenter Retrospective	84	N/A	N/A	1	CDK4/6 inhibitor+endocrine therapy	N/A	N/A	N/A	N/A
Carlino, 2022 [24]	Italy	Multicenter Retrospective	165	71/94	64	1	Palbociclib+AI or Fulvestrant	Not reached	19 vs 23 mo	N/A	31 mo
Douganiotis, 2022 [25]	Greece	Multicenter Retrospective	191	139/52	60	1	CDK4/6 inhibitor (Palbociclib/ribociclib/abemaciclib)+AI or Fulvestrant	Not reached	HER2 +2/ISH-negative: 20.8 mo HER2+1: 26.1 mo HER2-Zero: 40.2 mo	N/A	15 mo
Lapuchesky,2022 [27]	Argentina	Single-center Retrospective	186	64/122	55	Mixed	CDK4/6 inhibitor (Palbociclib/ribociclib/abemaciclib)+endocrine therapy	N/A	15.6 v 19 mo	N/A	N/A
Zattarin, 2023 [18]	Italy	Multicenter Retrospective	428	269/159	N/A	1	CDK4/6 inhibitor (Palbociclib/ribociclib/abemaciclib)+endocrine therapy	48.7 vs 58.3 mo	23.6 vs 32.3 mo	N/A	36 mo
Yildirim, 2023 [17]	Turkey	Multicenter Retrospective	204	66/138	58	Mixed	CDK4/6 inhibitor (Palbociclib/ribociclib)+AI (*n* = 115) CDK4/6 inhibitor (Palbociclib/ribociclib)+Fulvestrant (*n* = 89)	Not reached	19 vs 18 mo	72.7% vs 66.6%	22 mo
Sharaf, 2023 [19]	Jordan	Single-center Retrospective	257	143/114	49.9	Mixed	Ribociclib+AI or Fulvestrant	N/A	17.3 vs 22.2 mo	39.4% vs 52%	N/A
Mouabbi, 2023 [28]	USA	Single-center Cohort	1084	697/387	50	Mixed	CDK4/6 inhibitor (Palbociclib/ribociclib/abemaciclib)+endocrine therapy	First-line:32.4 mo vs 31.2 mo Second Line: 31.5 vs 24.9 mo	First-line:13 vs 11.6 mo Second Line: 7.3 vs 7.1 mo	N/A	17.9 mo

^*^AI: aromatase inhibitor, mo: months

**Table 2:** Newcastle-Ottowa Scores of Included Studies

First author, publication year	Publication type	Selection				Comparability	Outcome			Total score
		Representativeness	Selection of the non-exposed cohort	Ascertainment of exposure	Demonstration that outcome of interest was not present at start of study	Comparability of cohorts on the basis of the design or analysis	Assess-ment of outcome	Was follow-up long enough for outcomes to occur? (1‑year threshold)	Adequacy of follow-up of cohorts	
Bao, 2021 [26]	Full-text article	1	1	1	1	1	1	1	0	7
Bortot, 2021 [23]	Congress abstract	1	1	1	1	1	1	0	0	6
Carlino, 2022 [24]	Full-text article	1	1	1	1	2	1	1	1	9
Douganiotis, 2022 [25]	Full-text article	1	1	1	1	2	1	1	1	9
Lapuchesky,2022 [27]	Congress abstract	1	1	1	1	1	1	0	0	6
Zattarin, 2023 [18]	Full-text article	1	1	1	1	2	1	1	1	9
Yildirim, 2023 [17]	Full-text article	1	1	1	1	2	1	1	1	9
Sharaf, 2023 [19]	Full-text article	1	1	1	1	1	1	1	0	8
Mouabbi, 2023 [28]	Full-text article	1	1	1	1	2	1	1	1	9

### Association between HER2-low status and PFS

**Fig. 2:**
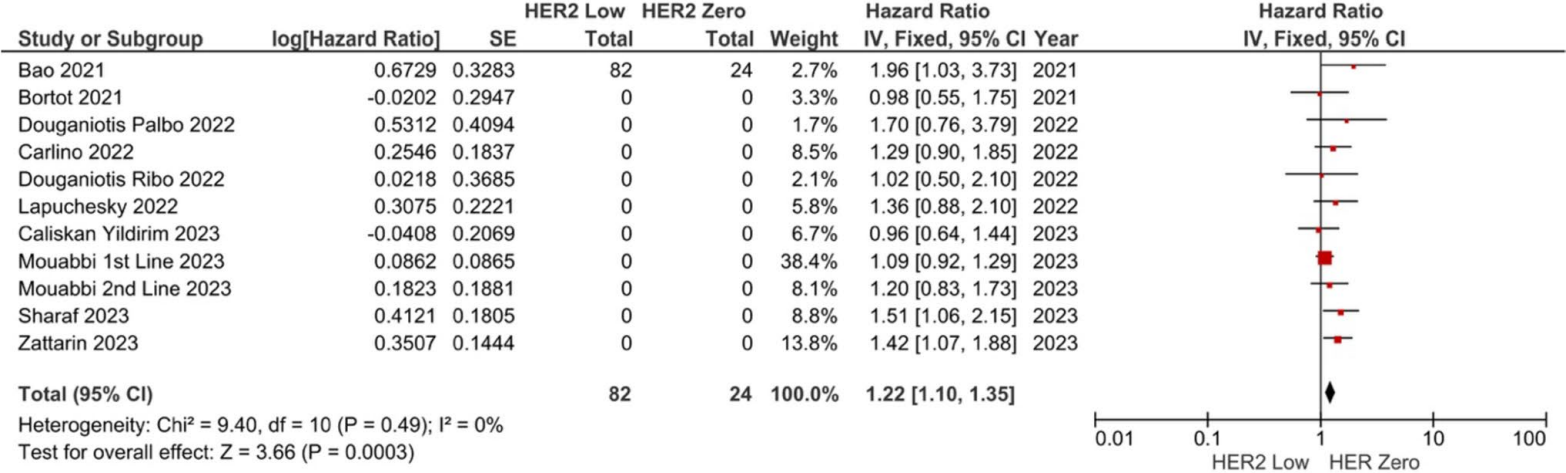
Meta-analysis of progression-free survival. Diamond indicates the pooled effect size

Six of nine studies reported no association between the HER2-low status and PFS with CDK 4/6 inhibitors [17, 23–25, 27, 28]. In the pooled analysis of nine studies, the risk of progression and/or death was higher in patients with the HER2-low tumors compared to HER2 zero (HR: 1.22, 95% CI 1.10–1.35, *p* < 0.001) (Fig. 2). The included studies had low degree of heterogeneity (I2 = 0%). Sensitivity analyses conducted by the subtraction of the individual studies demonstrated consistent results.

**Fig. 3:**
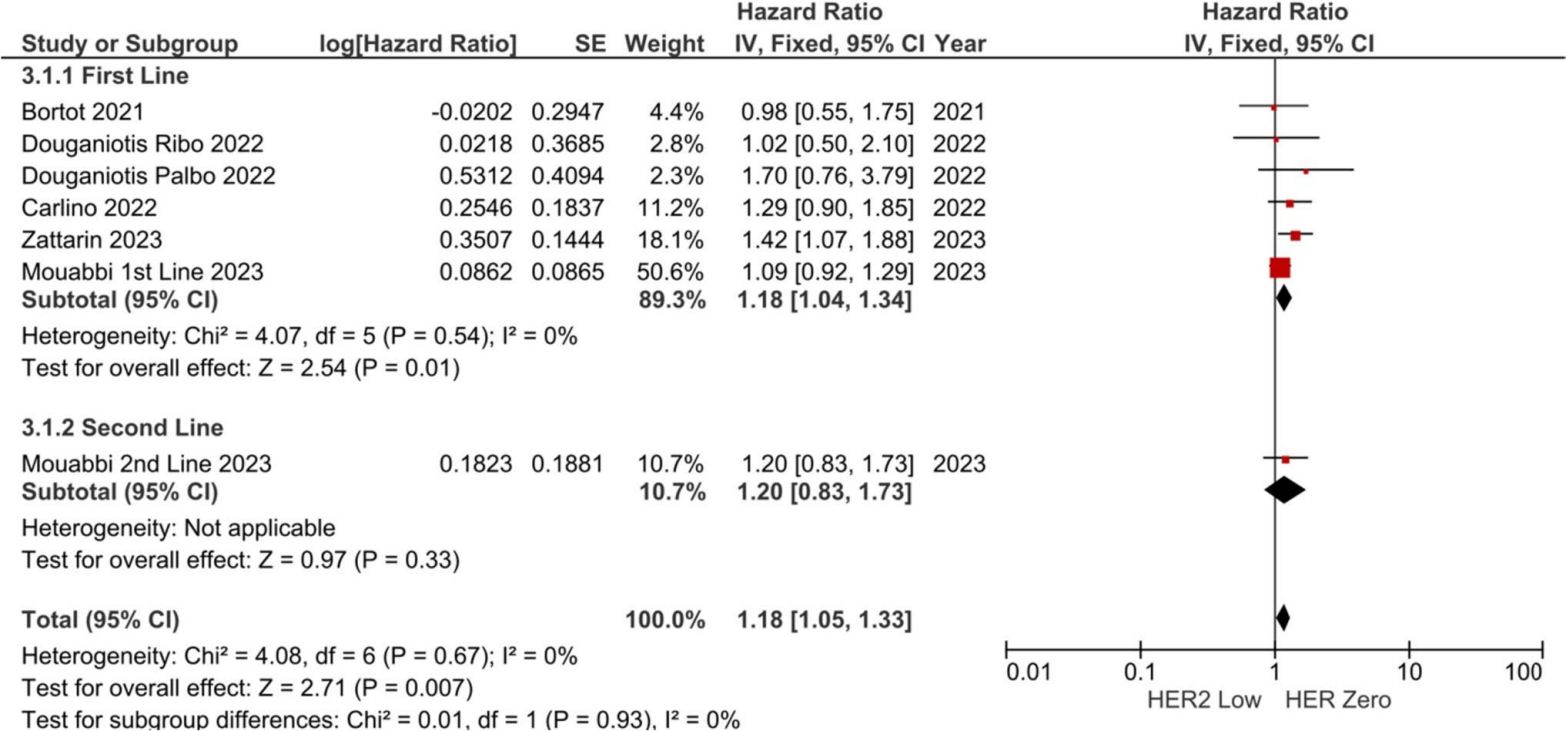
Subgroup analyses of PFS according to treatment line

Subgroup analyses were conducted according to the treatment line. The risk of progression and/or death was similar across the lines of treatment (1st-line HR: 1.18, 95% CI 1.04–1.34, *p* = 0.010, and 2nd-line HR: 1.20, 95% CI 0.83–1.73, *p* = 0.330, p-value for subgroup differences *p* = 0.930) (Fig. 3), although four studied did not have separate data for treatment lines and only one study specifically included patients treated in the second line.

### Association between HER2-low status and OS/ORR

**Fig. 4:**
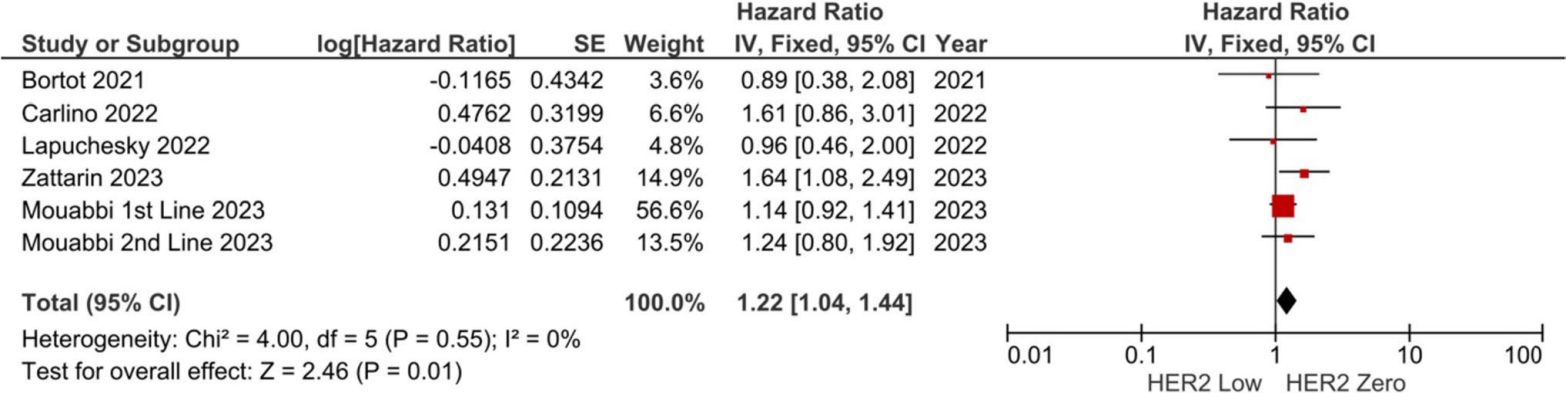
Meta-analysis of the overall survival. Diamond indicates the pooled effect size

**Fig. 5:**
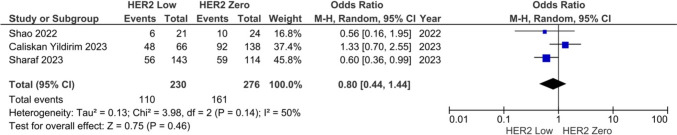
Meta-analysis of the overall response rate

A total of 5 and 3 studies were reported on OS and ORR, respectively. In the pooled analysis of five studies, the risk of death was higher in the HER2-low group compared to the HER2-zero group (HR: 1.22, 95% CI 1.04–1.44, *p* = 0.010) (Fig. 4). The included studies had low degree of heterogeneity (I2 = 0%), and sensitivity analyses conducted by the subtraction of the individual studies demonstrated consistent results. The pooled ORR with CDK 4/6 inhibitors was 47.8% in the HER2-low group and 58.3% in the HER2-zero group. The ORR was similar independent of the HER2-low status (HR: 0.80, 95% CI 0.44–1.44, *p* = 0.460) (Fig. 5). The meta-analysis for ORR had a high degree of heterogeneity (I2 = 50%).

## Discussion

In this meta-analysis of over 2700 patients, we observed significantly higher progression or death in patients with HR+HER2-low metastatic breast cancer compared to patients with HER2-zero tumors. Although the median follow-up was short, the risk of death was also higher in patients with HER2-low expression. The ORR was similar across the HER2-low and HER2 groups, although the sample size was smaller for this analysis. The PFS analyses were consistent across the treatment line.

The characteristics of patients who had early progression with CDK 4/6 inhibitors is a critical research field. While earlier data suggested several clinical features like visceral metastases and ECOG status, molecular biomarkers like RB1 and CCNE1 were also associated with a higher risk of progression [1, 29–33]. In addition, tumor molecular subtyping via PAM50 (prosigna) was also associated with the efficacy of CDK 4/6 inhibitors [34, 35]. In the study by Prat et al., patients with HER2-enriched HR+breast cancer had early risk progression risk with palbociclib [36]. In contrast, a similar pattern was absent in patients treated with ribociclib. However, the PAM50 (prosigna) assay is not routinely available in daily practice due to financial reasons and primarily licensed for the early breast cancer. Considering the financial limitations of RNA-based profiling for HER2 enrichment, evaluation of HER2-low status by immunohistochemistry could be a surrogate for the activation of the ErbB2 pathway in patients treated with CDK 4/6 inhibitors. Furthermore, it was previously demonstrated that HER2-low tumors had higher ESR1 [37] and AKT expressions [38], features associated with resistance to CDK 4/6 inhibitors. Therefore, using HER2-low status as an efficacy biomarker in patients treated with CDK 4/6 inhibitors could be beneficial due to the strong biological rationale.

Despite the strong interest, the data on the association between HER2-low status and CDK 4/6 inhibitor efficacy are still controversial. A similar problem was present with the survival outcomes with early HER2-low breast cancer, with studies with contrasting results also available [39–42]. One of the main reasons regarding this issue could be the problems and variability with HER2-low case definition. There is significant variability across reading pathologists regarding the HER2-low status [43]. Additionally, it was demonstrated that HER2-low status could vary between the primary tumor and the metastasis [44, 45]. However, the source of the HER2-low definition (primary vs metastasis) was absent in the included studies in the meta-analysis [46]. Further research on the prognostic role of HER2-low status should ideally evaluate interobserver variability for case definition and report on the tissue in which the HER2-low status was evaluated.

The present meta-analysis is subject to several limitations. First, most of the available studies were retrospective and had limited sample sizes. The study cohorts were also heterogeneous regarding the treatment line and endocrine treatment partner limiting the ability to conduct subgroup analyses with adequate power. The follow-up time was short in most studies, limiting the reliability of overall survival results. The adjustments according to additional clinical parameters were absent in most studies. Lastly, due to the retrospective nature of most studies, causality regarding the effects of HER2-low status on survival outcomes could not be assured, and we opted to use the term association instead of effect in our reporting. However, despite these limitations, we observed a negative effect of low-level HER2 expression on survival outcomes in a pooled cohort of over 2700 patients. If our results are supported by prospective studies with longer follow-ups, the patients with advanced HR+HER2-low breast cancer could be candidates for novel combination approaches to improve outcomes with CDK 4/6 inhibitors.

## Conclusion

In conclusion, the available evidence demonstrates a significantly higher risk of progression or death with CDK 4/6 inhibitors in HER2-low tumors. While the CDK 4/6 inhibitor plus endocrine therapy is the standard of care independent of the HER2-low status, further research is needed to improve outcomes in patients with HR+HER2-low tumors.

## Data Availability

The datasets used and/or analyzed during the current study are available from the corresponding author on reasonable request.
